# Etiological influences on the stability of autistic traits from childhood to early adulthood: evidence from a twin study

**DOI:** 10.1186/s13229-017-0120-5

**Published:** 2017-02-17

**Authors:** Mark J. Taylor, Christopher Gillberg, Paul Lichtenstein, Sebastian Lundström

**Affiliations:** 1 Department of Medical Epidemiology and Biostatistics, Karolinska Institutet, Nobels väg 12A, 17177 Stockholm, Sweden; 2 Gillberg Neuropsychiatry Centre, Institute of Neuroscience and Physiology, University of Gothenburg, Kungsgatan 12, 41119 Gothenburg, Sweden; 3 Centre for Ethics, Law, and Mental Health, Institute of Neuroscience and Physiology, University of Gothenburg, Rågården, Hus 1, SU-Östra sjukhuset, 41685 Gothenburg, Sweden

**Keywords:** Autism, Twin study, Genetics, Stability, Adulthood

## Abstract

**Background:**

Autism spectrum disorders (ASD) are persistent and lifelong conditions. Despite this, almost all twin studies focus on childhood. This twin study investigated the stability of autistic traits from childhood to early adulthood and explored the degree to which any stability could be explained by genetic or environmental factors.

**Methods:**

Parents of over 2500 twin pairs completed questionnaires assessing autistic traits when twins were aged either 9 or 12 years and again when twins were aged 18. Bivariate twin analysis assessed the degree of phenotypic and etiological stability in autistic traits across this period. Genetic overlap in autistic traits across development was also tested in individuals displaying a broad ASD phenotype, defined as scoring within the highest 5% of the sample.

**Results:**

Autistic traits displayed moderate phenotypic stability (*r* = .39). The heritability of autistic traits was 76–77% in childhood and 60–62% in adulthood. A moderate degree of genetic influences on childhood autistic traits were carried across into adulthood (genetic correlation = .49). The majority (85%) of the stability in autistic traits was attributable to genetic factors. Genetic influences on autistic traits were moderately stable from childhood to early adulthood at the extremes (genetic correlation = .64).

**Conclusions:**

Broad autistic traits display moderate phenotypic and etiological stability from childhood to early adulthood. Genetic factors accounted for almost all phenotypic stability, although there was some phenotypic and etiological instability in autistic traits. Thus, autistic traits in adulthood are influenced by a combination of enduring and unique genetic factors.

**Electronic supplementary material:**

The online version of this article (doi:10.1186/s13229-017-0120-5) contains supplementary material, which is available to authorized users.

## Background

Autism spectrum disorders (ASD) are neurodevelopmental disorders characterized by atypical social communication and behavioral inflexibility [1]. There is now increasing evidence that characteristic autistic traits are present to varying degrees throughout the population [2]. Furthermore, both clinical ASD [3, 4] and autistic traits [5–7] are highly heritable. One recent study reported that the genetic factors associated with a gold standard research diagnosis of ASD also influenced broader autistic traits [4]. Thus, ASD appears to be linked with milder autistic traits, with individuals with ASD representing the extreme end of a continuous distribution of autistic traits.

Under present diagnostic criteria, symptoms of ASD are required to have presented from an early period of development [1]; indeed, symptoms of ASD appear discernable early in life [8, 9]. This was even initially noted by Kanner [10] in his 1943 report of eleven children with ASD, who noted that parents of the children had observed ASD symptoms from when their children were infants. Subsequently, he published a report following a subset of these ASD cases up. He reported that for many of them, their ASD symptoms were still present when they were adults [11]. This has been corroborated in more recent years by larger clinical studies [12–16], thus emphasizing the stable nature of ASD. ASD in adulthood may, in some instances, be associated with negative outcomes, such as unemployment [17], earlier mortality [18, 19], and poorer daily living skills [20], stressing the need for a better understanding of the persistence of ASD into adulthood.

Reasonably little is known about whether broader autistic traits are similarly stable from childhood into adulthood. Such a question is of relevance, since similar stability of ASD and autistic traits is another strand of evidence regarding the continuous nature of ASD. Three studies have focused on the stability of autistic traits across childhood. The first focused on two US-based samples of twins aged 3–18. Across a 5-year follow-up period, autistic traits were stable, albeit small decreases were also observed [21]. The second study, of a UK-based cohort, reported a very small decrease in autistic traits in males between the ages of 7–15. This aside, autistic traits were stable, regardless of initial severity level [22]. Finally, a UK-based twin study reported a strong correlation between autistic traits assessed between ages 8–12 [23]. Only one study has investigated the stability of autistic traits from childhood to adulthood; a weak correlation (*r* = .17) was reported between autistic traits assessed by parents using the Child Behavior Checklist at age 2 and the self-rated Autism Spectrum Quotient in adulthood [24]. The reasonably low correlation was likely driven by the use of two different measures administered to different raters over a wide period.

Further, little is known about the stability of the etiology of autistic traits between childhood and adulthood. The few existing twin studies of adults report that autistic traits are slightly less heritable in adulthood than during childhood [25–28], yet it is not clear whether these causal influences on autistic traits are carried across from childhood. One twin study reported that the strong correlation between autistic traits across childhood was largely attributable to genetic influences that were common to all assessments between ages 8 and 12 [23]. It is not known whether the same pattern of stability extends into adulthood.

This study thus aimed to investigate the stability of autistic traits from childhood to early adulthood. Specifically, we aimed to elucidate the extent of phenotypic and etiological stability in autistic traits across this period, as well as establish the degree to which any stability in autistic traits could be explained by genetic or environmental influences. ASD appears to be moderately stable into adulthood as a dichotomous diagnosis and highly stable across childhood as a continuous trait; thus, we hypothesized that there would be a significant degree of stability in autistic traits from childhood to early adulthood.

## Methods

### Participants

Data were collected from participants in the Child and Adolescent Twin Study in Sweden (CATSS [29]). All twins born in Sweden since 1 July 1992 were contacted in connection with the twins’ ninth or twelfth birthday, with a response rate of 80% (879 pairs aged 9 [mean = 9.08, SD = 0.27], 1822 pairs aged 12 [mean = 12.05, SD = 0.13]). Of those invited to participate in CATSS at age 18, 59% have responded (mean age = 18.31, SD = 0.21). Data were available at both ages for 2701 twin pairs with known zygosity. The response rate of 59% of participants who had taken part in CATSS at ages 9 or 12 who were invited to participate at age 18 was lower than the response rate at ages 9/12, suggesting some attrition. The participating and non-participating samples at age 18, however, were comparable with regard to ASD prevalence. Of the included pairs at age 18, 34 (1% of the sample) had received an ASD diagnosis according to the Swedish National Patient Register. Of the 9858 non-participating pairs, 106 individuals had ASD (1% of the sample). Thus, the participating and non-participating samples were comparable with regard to ASD prevalence. Table 1 shows additional demographic characteristics for the participating and non-participating families, suggesting that the two samples were reasonably comparable.

**Table 1:** Descriptive statistics

Sample characteristics								
	Sex		Zygosity		Highest parental education level			
	% Male	% Female	% MZ	% DZ	% Compulsory School	% Upper Secondary School	% College/university	% Other
Participants in CATSS-18	47%	53%	35%	65%	M: 5% F: 12%	M: 45% F: 47%	M: 46% F: 38%	M: 4% F: 3%
Non-responders	52%	48%	28%	72%	M: 4% F: 9%	M: 45% F: 53%	M: 48% F: 36%	M: 3% F: 2%
Descriptive statistics								
A-TAC scale	*N* Items		Range		Mean (SD)		Cronbach’s α	Skew
Full scale, age 9/12	12		0–12		0.34 (0.86)		.74	5.50
Full scale, age 18	12		0–11		0.73 (1.07)		.70	3.18

*M* highest maternal education level, *F* highest paternal education level, *A-TAC* Autism-Tics, AD/HD and other Comorbidities inventory

Exclusions were conducted for any medical condition that may have influenced the etiology and phenotypic expression of autistic traits, including known brain injuries (*N* = 31 pairs), chromosomal disorders (*N* = 4 pairs), and/or epilepsy (*N* = 30 pairs). Sixty-four pairs were removed, leaving a sample of 2637 pairs (386 monozygotic (MZ) male, 430 dizygotic (DZ) male, 539 MZ female, 447 DZ female, and 835 DZ opposite-sex). Sixty-four percent of same-sex pairs born up until 2001 had their zygosity ascertained using a panel of five single-nucleotide polymorphisms. The remainder was assessed using an algorithm of five questions about twin similarity; only twins with more than 95% probability of being correctly classified using this method were included.

CATSS has ethical approval from the Karolinska Institutet Ethical Review Board. Informed consent was obtained from all families prior to participation.

### Measures

Autistic traits were assessed at both ages using the Autism-Tics, AD/HD and other Comorbidities inventory (A-TAC [30]). The A-TAC was designed as a brief screening tool for neurodevelopmental disorders and is a fully structured interview of 96 questions. ASD was assessed by 12 items at both ages corresponding to DSM-IV criteria for ASD, making the measure shorter than alternatives such as the Social Responsiveness Scale or Autism Spectrum Quotient [31]. At ages 9/12, the A-TAC was administered by an interviewer over the telephone, who used a computerized version of the interview and recorded parental responses. At age 18, parents assigned ratings to each question themselves using an internet-based version of the assessment. All questions were answered “yes” (scored as 1), “yes, to some extent” (0.5), or “no” (0). The maximum possible score was 12 at both ages. The items administered are shown in Additional file 1. The A-TAC is freely available at www.gnc.gu.se and as an appendix to a previous paper [31].

As shown in Table 1, the A-TAC had acceptable internal consistency at both ages (.70–.74). The measure has been extensively validated. Cross-sectionally, the 12 A-TAC items have an area under the curve of 0.96 and sensitivity of 0.86 and specificity of 0.94 for the identification of ASD (according to reanalysis of the data from the paper by Larson et al.) [31]. The A-TAC has also been followed up longitudinally, with excellent psychometric properties reported [32]. Strong psychometric properties for the A-TAC were also reported in an independent sample [33]. While the A-TAC has not been validated for use with adults, ASD cases identified using the Swedish National Patient Register (*N* = 34) scored significantly higher (mean = 4.07, which was very close to the suggested cutoff of 4.5) than controls (mean = 0.64), t_33.07_ = −6.97, *p* < .001. As one would expect from a valid measure of autistic traits, males (mean = 0.81) scored significantly higher than females (mean = 0.66) on the A-TAC at age 18, t_2097.77_ = 3.16, *p* < .01. The A-TAC also correlates to a moderate degree with other validated scales assessing behaviors characteristic of ASD, for example a correlation of .43 with the Child Behavior Checklist social problems subscale has been reported [34].

### Data analysis

To increase statistical power, data at the age of first assessment (age 9 or 12) were combined, rather than analyzing data separately for twins aged 9 and twins aged 12 at first assessment. There was also no significant difference in the magnitude of the correlation between A-TAC scores at the two ages when the sample was split into those who responded at age 9 and those who responded at age 12 (contact the first author for details).

#### Data preparation

The A-TAC was skewed at both ages (see Table 1 for skew values, and Additional file 2 for histograms) and was thus log transformed prior to analysis. The effects of sex and age were, where present, regressed out of the means, as is standard behavioral genetic procedure [35]. Analyses of continuous scores were conducted on scores standardized by sex and age.

#### Phenotypic analyses

Phenotypic stability in autistic traits was ascertained from phenotypic correlations (*r*_ph_) between the A-TAC scales at each age, estimated from a constrained saturated model in which means, variances, and phenotypic correlations were equated across twin order.

The twin design capitalizes on the fact that MZ twins are genetically identical, compared with DZ twins, who share approximately 50% of their segregating DNA code. In comparing the relative similarity of MZ and DZ twins on a trait, it is possible to deduce the degree of genetic and environmental influence on a single trait, as well as the extent to which these influences overlap across two or more traits.

Twin correlations are the foundations of twin models. One twin’s score on a measure was first correlated with their co-twin’s score on the same measure (cross-twin correlations). In ascertaining these statistics by zygosity, one gains an indication of the extent of genetic and environmental influence on a trait. Owing to the genetic similarity of MZ twins relative to DZ twins, additive genetic influences (A) are indicated when the MZ correlation exceeds the DZ correlation. Any discrepancy between MZ twins is assumed to be due to nonshared environment (E), which denotes environmental factors that create differences between twins in a pair. The opposite, shared environment (C), is indicated by DZ correlations in excess of half the MZ correlations. If the DZ correlation is less than half the MZ correlation, then non-additive genetic influences (D) may be in operation.

Cross-trait cross-twin correlations were then estimated. One twin’s A-TAC score at age 9/12 was correlated with their co-twin’s A-TAC score at age 18. These statistics denote the degree to which genetic or environmental factors account for the covariance between two traits, in this instance the phenotypic stability in autistic traits. They can be interpreted in a similar manner to cross-twin correlations, with the exception that E is denoted by an MZ cross-trait cross-twin correlation that is lower than the phenotypic correlation. All twin correlations were estimated from a constrained saturated model, as described above.

#### Bivariate twin model

Twin models were fitted to estimate the proportion of each trait due to A, D or C, and E (E encompasses measurement error). The association between two phenotypes can then be investigated using bivariate Cholesky decompositions. A transformation of the Cholesky, the correlated factor solution, was fitted to data in order to estimate *etiological correlations* between the A, C or D, and E variance components, which denote the degree of causal overlap between two traits (in this case, etiological stability). Estimates of 1 indicate total overlap, while estimates of 0 suggest no overlap. One can then calculate the proportion of the phenotypic correlation attributable to A, C or D, and E.

Since C and D are confounded in the classical twin design, ACE or ADE models were fitted based on the twin correlations. The twin design assumes that variance is equal across zygosity, yet this assumption can be violated when sibling contrast effects arise. These effects refer to the instance whereby one parent’s rating of one twin causes them to ascribe lower ratings to their co-twin. The impact of such effects is to increase DZ variance relative to MZ variance, which was the case for our data (see Additional file 3). Models thus estimated sibling contrast effects by adding a causal pathway (termed *s*) between one twin’s phenotype and their co-twin’s phenotype. Cross-twin correlations could not be collapsed across same-sex twin pairs (see Additional file 1); thus, we tested *quantitative sex limitation*, allowing variance components and etiological correlations to differ in magnitude across sexes. ADE or ACE models were compared to a series of reduced models: an ADE/ACE model with equal etiological correlations across sex (scalar model), ADEs/ACEs with all sex differences dropped (homogeneity model), and then further reduced models which removed individual variance components. The fit of these models was compared with the ACE/ADE model using the likelihood ratio test; each model was summarized by the -2LL fit statistic. Differences in -2LL between two models are *χ* ^2^ distributed, with degrees of freedom equal to the discrepancy in number of parameters between two models. Significant *p* values indicated that a given model fit the data significantly more poorly than the unreduced ACE/ADE model.

Analyses were conducted in the OpenMx [36] package of R [37].

#### Extremes analysis

DeFries-Fulker analysis was used to test genetic overlap between extreme scores on the A-TAC at each age. This method was chosen over alternatives since it is suitable for skewed data [38] and has been widely used for bivariate research questions in twin studies.

Extreme scorers (termed *probands*) were selected on the basis of scoring within the highest 5% of the A-TAC ASD scale at each age. This cutoff was used to maximize statistical power while also ensuring that probands displayed reasonably severe scores. This corresponded to a score of at least 2.5 at ages 9/12 (*z*-score = 2.46) and 3 or above at age 18 (*z*-score = 2.05).

A more thorough explanation of DeFries-Fulker analysis is provided in Additional file 4. Briefly, it is a regression-based procedure. Univariate procedures estimate group heritability, which refers to the degree to which genetic factors influence the mean difference between a proband group and the rest of the sample [39]. Group heritability was estimated using a regression equation for predicting scores of co-twins of probands from proband scores and zygosity. Bivariate DeFries-Fulker analysis is an extension of this procedure, which estimates bivariate heritability, the extent to which genetic influences on extreme scores on one trait influence continuous variation on the second trait [40]. In the bivariate case, the regression equation predicts co-twin scores on the second measure of interest from proband scores on the measure used to select probands and zygosity. Performing univariate and bivariate DeFries-Fulker analysis allows calculation of a genetic correlation between extreme scores on two traits [41].

DeFries-Fulker analyses were all conducted in R [37].

## Results

Descriptive statistics are given in Table 1.

Phenotypic correlations were similar across sexes (males = .38 [.34–.41]; females = .41 [.36–.45]). Collapsed across sexes, the phenotypic correlation was .39 (.36–.41).

Twin correlations are given in Table 2. At age 9/12, MZ correlations (.65–.70) exceeded DZ correlations (.20–.26) in both sexes, suggesting genetic influences on autistic traits in childhood. The same pattern was true at age 18; MZ correlations (.57–.59) were all greater than DZ correlations (.21–.25). At age 18, MZ correlations were substantially lower than 1, suggesting a larger nonshared environmental component in early adulthood. Cross-trait cross-twin correlations were all higher for MZ twins (.32 in both sexes) than DZ twins (.09–.16), suggesting that most of the phenotypic correlation between autistic traits at age 9/12 and age 18 was due to additive genetic factors.

**Table 2:** Twin correlations

	MZM	DZM	MZF	DZF	DZOS
Cross-twin, age 9/12	.70 (.65-.74)	.21 (.13–.28)	.65 (.60–.69)	.20 (.12–.28)	.26 (.20–.32)
Cross-twin, age 18	.59 (.52–.64)	.21 (.11–.30)	.57 (.51–.62)	.24 (.15–.33)	.25 (.17–.32)
Cross-trait cross-twin	.32 (.27–.37)	.10 (.03–.17)	.32 (.26–.37)	.09 (.02–.16)	.16 (.10–.21)

95% confidence intervals are shown in parentheses

*Cross-twin, age 9/12* correlation of one twin’s A-TAC score at age 9/12 with their co-twin’s A-TAC score at age 9/12, *Cross-twin, age 18* correlation of one twin’s A-TAC score at age 18 with their co-twin’s A-TAC score at age 18, *Cross-trait, cross-twin* correlation of one twin’s A-TAC score at age 9/12 with their co-twin’s A-TAC score at age 18, *MZM* monozygotic male, *DZM* dizygotic male, *MZF* monozygotic female, *DZF* dizygotic female, *DZOS* dizygotic opposite-sex

DZ correlations were lower than half the MZ correlations, suggesting non-additive genetic factors. Consequently, an ADE model with quantitative sex limitation was fitted. Fit statistics are shown in Table 3. Dropping quantitative sex differences in the etiological correlations did not significantly reduce the fit of the model (*p* = .21), although variance components could not be collapsed across sexes (*p* < .001). Non-additive genetic parameters could all be dropped (*p* = .27), although no other components could be dropped (see Table 3).

**Table 3:** Twin model fit statistics

Full model								
Model	-2LL	Parameters	df	AIC	BIC	Δ*χ* ^2^	Δdf	*p*
Saturated	25,044.31	70	9494	6056.31	−49,743.70	–	–	–
ADEs	25,155.82	28	9536	6083.82	−49,963.04	111.51	42	<.001
Nested models								
Model	-2LL	Parameters	df	AIC	BIC	Δ*χ* ^2^	Δdf	*p*
ADE scalar	25,160.31	25	9539	6082.31	−49,982.19	4.84	3	.21
ADE hom.	25,220.50	15	9549	6122.50	−50,000.77	64.68	13	<.001
AE scalar	25,165.81	20	9544	6077.81	−50,016.07	9.99	8	.27
AE scalar	25,208.87	14	9550	6108.87	−50,020.27	53.05	14	<.001
E scalar	26,053.90	9	9555	6943.90	−49,214.63	898.08	19	<.001

Statistics for the ADEs model are in comparison with the saturated model. Statistics for all other models are in comparison to the ADEs model. Saturated model: model of the observed data, allowing means and variances to differ across twin order and zygosity. All other models constrain means and variances to be equal across twin order and zygosity

*-2LL* fit statistic, which is -2*log-likelihood of the data, *df* degrees of freedom, *AIC* Akaike’s Information Criteria, *BIC* Bayesian Information Criteria, *Δχ* ^*2*^ change in -2LL between two models, distributed, χ^2^, *Δdf* change in degrees of freedom between two models, *A* additive genetic influence, *D* non-additive genetic influence, *E* nonshared environmental influence, *s* sibling interaction effects, *hom*. homogeneity model, which collapsed all estimates across sex

**Fig. 1:**
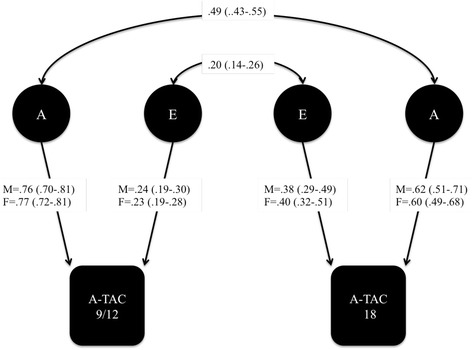
Bivariate twin model showing parameter estimates. The *single-headed arrows* connecting the circular (latent) variables to the observed variables show the proportion of each trait explained by A and E. The *double-headed arrows* connecting the latent variables show the correlation between these variables. Sibling interaction pathways not shown. *A-TAC* Autism-Tics, AD/HD and other Comorbidities Inventory, *A* additive genetic influence, *E* nonshared environmental influences

Figure 1 shows the estimates from the AE model. Most variance in autistic traits at age 9/12 was additive genetic (male = .76 [.70–.81]; female = .77 [.72–.81]), with the remainder nonshared environmental (male = .24 [.19–.30]; female = .23 [.19–.28]). Additive genetic influences decreased significantly in females at age 18 (male = .62 [.51–.71]; female = .60 [.49–.68]), with nonshared environment increasing (male = .38 [.29–.49]; female = .40 [.32–.51]). There were sibling contrast effects in autistic traits at age 9/12 (male = −.08 [−.09/−.06]; female = −.13 [−.15/−.11]; opposite-sex twins = −.07 [−.08/−.05]) and age 18 (male = −.04 [−.05/−.01]; female = −.02 [−.05/.00], opposite-sex twins = −.04 [−.06/−.02]). There was moderate genetic stability in autistic traits from childhood to early adulthood, with an additive genetic correlation of .49 (.43–.55). The nonshared environmental correlation was also significant (.20 [.14–.26]). Eighty-five percent of the phenotypic correlation between A-TAC scores at age 9/12 and age 18 was due to additive genetic factors. The remaining 15% was accounted for by nonshared environmental influences.

Results of DeFries-Fulker analyses are given in full in Additional file 5. Group heritability was moderate at age 9/12 (.59 [.28–.59]) and age 18 (.69 [.36–.69]). The genetic correlation between extreme autistic traits from childhood to early adulthood was .64, suggesting considerable genetic stability across development.

## Discussion

This longitudinal study sought to investigate the degree to which autistic traits are phenotypically and etiologically stable from childhood through to adulthood, thus building upon an existing twin study of middle childhood [23]. There was a moderate degree of stability in autistic traits across the 6–9-year period studied. Genetic factors that were common to both ages explained almost all of the stability in autistic traits, albeit the genetic correlation between autistic traits across development was some way short of 1. Thus, moderately overlapping genetic influences from childhood to adulthood appear to be a key factor in explaining the stability of autistic traits across this period.

The stability of autistic traits from childhood to early adulthood observed here does appear to be weaker than the stability of autistic traits reported across childhood [21–23]. Of note, however, the correlation between childhood and early adult autistic traits reported here was more than double the correlation previously reported [24]. Differences in measurement and ages of assessment are the most likely explanation for these discrepant findings. The previous study assessed autistic traits using two different measures (the Child Behavior Check List in childhood and the Autism Spectrum Quotient in adults); the first of which was administered at age 2. We used the same measure, initially administered at ages 9 or 12 rather than age 2 and covered a narrower age range.

Our findings instead appear to be reminiscent of clinical diagnoses of ASD, which have been shown to display moderate stability from childhood through to adulthood [12–16]. In one of these studies, individuals who met criteria for Asperger syndrome were assessed over two decades. There was a significant decrease in the number of individuals meeting criteria as adults, yet diagnoses were more stable in more severe cases [15]. In our study, the phenotypic stability of autistic traits was indeed higher at the quantitative extreme. Thus, our findings seem to present a similar pattern of stability for autistic traits to clinical diagnoses.

The similar stability of autistic traits and ASD into early adulthood opens up two implications. First, our findings add weight to the hypothesis that ASD lies on a continuum with autistic traits. Previous studies suggest similar etiology of autistic traits and ASD [4–7, 42]. In particular, correlations assessed in the full sample and at the extreme were similar, suggesting continuity in the observed stability across milder traits and more severe manifestations. Second, the dimensional nature of autistic traits is also apparent in early adulthood, reflecting other studies of autistic traits in adults [43–46]. Thus, dimensional assessments of ASD are a viable approach to genetic research into ASD not only in children but also adults.

That there was some instability in autistic traits from childhood to early adulthood is particularly worth highlighting, however. It may thus be that, to a degree, different genetic factors influence autistic traits in middle childhood and early adulthood. The paucity of genetic studies focusing on adults with ASD makes speculation about which genetic factors may differ across development challenging [47]. An extension to our study could therefore be to investigate whether polygenic scores for ASD differ across childhood and adulthood, which would be another line of evidence testing the degree of genetic stability in ASD from childhood to adulthood. The reasonably modest degree of phenotypic stability in our study also raises the possibility that the expression of autistic traits differs between childhood and early adulthood. Measures based on DSM-IV criteria, which are based on children, may not fully capture age-dependent expressions of autistic traits. Indeed, little is known about autistic traits in adulthood beyond the fact that they are present [43–46]. Thus, an important future direction for research is to better understand the nature of autistic traits in adults, which will benefit future endeavors to understand the genetics and stability of autistic traits into later periods of adulthood.

It is also worth highlighting the correlation between nonshared environmental influences across development, which was higher than reported in a previous twin study of the stability of autistic traits [23]. This is potentially surprising given that nonshared environmental factors seldom appear to have an enduring effect on behavioral phenotypes across development [48]. It is worth arguing, however, that the nonshared environmental factors linked with autistic traits have largely included perinatal and prenatal factors [49, 50]. One would not necessarily expect the effect of these factors to change over time, which could account for the reasonably high nonshared environmental correlation.

Our study was not free of limitations. While the A-TAC can be used to assess specific autistic trait domains, such as social problems and inflexible behaviors, the number of items corresponding to each domain was small in this study. Symptoms of ASD may display somewhat disparate developmental courses [51]. It has been suggested that autistic traits are “fractionated,” in that individual symptom domains may be underpinned by partly independent etiological factors [52, 53]. Thus, it will be important for future longitudinal twin research to test whether the stability of autistic traits differs according to individual trait domains.

We also note that the switch from administering the A-TAC over the telephone at age 9/12 to using an internet-based questionnaire at age 18 may have led to potential biases in our results. Mean scores were higher at age 18, for example. The difference in measure administration could also have attenuated the phenotypic correlation. Nonetheless, we note that the internal consistency was similar for both versions of the A-TAC (see Table 1), while a study comparing internet- and telephone-based assessments of other psychiatric phenotypes found similar internal consistency and test-retest reliability for both methods of administration [54].

We only employed assessments of autistic traits at two ages (9/12 and 18), which is a likely source of some instability in this study. For example, some of the etiology of autistic traits at age 18 could be due to innovative causal influences that emerge at an age we did not cover, such as the adolescent period. Future studies should seek to bridge this gap. In addition, while we examined the stability of autistic traits in the highest scoring 5% of our sample, approximating a broader ASD phenotype, we did not have sufficient statistical power to analyze the highest scoring 1% (mirroring the prevalence of ASD) or clinical cases. Previous twin studies, however, demonstrate consistent heritability of autistic traits of differing degrees [6, 7], while a meta-analysis recently demonstrated that heritability of autistic traits was consistent across a wide number of cutoffs [55]. Thus, we are confident that our findings extend to higher scoring groups than we were able to study here.

Future studies should also seek to obtain ratings of autistic traits from multiple sources, such as teachers and self-reports, to obtain a more complete picture of the stability of autistic traits from childhood to adulthood. One may question whether our findings derived from twins extend to singletons, although autistic traits have not been shown to differ between twins and singletons [56]. Interpretation of our results should be taken in light of the fact that twin models fitted to the data were significantly poorer fits to the data than a saturated model of the observed data. Poor model fits can arise from a number of factors, such as larger samples leading to small deviations in model fit showing up as significant [6] and from skewed data [57].

## Conclusions

To our knowledge, this is the first twin study to investigate the stability of autistic traits from childhood to early adulthood. Phenotypic stability in autistic traits was moderate and largely attributable to enduring genetic factors across development. The overlap in genetic influences across development was not total, however. Thus, one could conclude that autistic traits are initially caused by a given set of genetic and environmental factors in childhood, which persist to a certain degree, yet variation in autistic traits in young adults may be better understood through a combination of enduring and innovative genetic influences across development. Such age-specific effects are potentially important to account for when designing future studies on the expression and etiology of autistic traits.

## Additional files

Additional file 1: A-TAC items. The 12 A-TAC questions administered at both ages. (PDF 66 kb)
Additional file 2: Distribution of A-TAC scores. Histograms showing the distribution of the untransformed and transformed A-TAC scales. (PDF 101 kb)
Additional file 3: Saturated model fitting. Table showing the results of saturated model fitting used to test assumptions and sex differences. (PDF 117 kb)
Additional file 4: Description of DeFries-Fulker analytic method. Detailed outline of the DeFries-Fulker analytic procedure used to analyze extreme scoring groups. (PDF 266 kb)
Additional file 5: DeFries-Fulker analysis results. Three tables showing the full results of DeFries-Fulker analysis of the extremes, including descriptive statistics, univariate results, and bivariate results. (PDF 88 kb)

## Abbreviations

A: Additive genetic influence
C: Shared environmental influence
D: Non-additive genetic influence
DZF: Same-sex female dizygotic twins
DZM: Same-sex male dizygotic twins
DZOS: Opposite-sex dizygotic twins
E: Nonshared environmental influence
MZF: Same-sex female monozygotic twins
MZM: Same-sex male monozygotic twins
r_A_: Additive genetic correlation
r_C_: Shared environmental correlation
r_D_: Non-additive genetic correlation
r_E_: Nonshared environmental correlation
s: Sibling contrast effects
