# Supplementary material for: Etiological influences on the stability of autistic traits from childhood to early adulthood: evidence from a twin study
Source: Mol Autism. 2017 Feb 17;8:5. doi: 10.1186/s13229-017-0120-5 (PMC5351180; doi:10.1186/s13229-017-0120-5)
Supplement: Additional file 1: — A-TAC items. The 12 A-TAC questions administered at both ages. (PDF 66 kb) [file 13229_2017_120_MOESM1_ESM.pdf]

A-TAC Items

1. Does the twin have difficulties expressing emotions and reactions with facial gestures, prosody, or body language?

2. Does the twin have difficulties making and keeping friends?

3. Is the twin interested in sharing joy, interests, and activities with others?

4. Does the twin have difficulties being with other people if it is not on his/her terms?

5. Was the twin's language development delayed?

6. Does the twin have difficulties sustaining a conversation with others?

7. Does the twin like to repeat words and expressions or does s/he use words in a way other people find strange?

8. Does the twin have difficulties with imitating other people or playing charades?

9. Does the twin get absorbed by his/her interests in such a way as being repetitive or too intense?

10. Does the twin get absorbed by routines in such a way as to produce problems for his/herself or for others?

11. Does the twin have specific body movements that appear automatically when he/she is happy or upset?

12. Does the twin get absorbed by details?
