# Supplementary material for: Etiological influences on the stability of autistic traits from childhood to early adulthood: evidence from a twin study
Source: Mol Autism. 2017 Feb 17;8:5. doi: 10.1186/s13229-017-0120-5 (PMC5351180; doi:10.1186/s13229-017-0120-5)
Supplement: Additional file 2: — Distribution of A-TAC scores. Histograms showing the distribution of the untransformed and transformed A-TAC scales. (PDF 101 kb) [file 13229_2017_120_MOESM2_ESM.pdf]

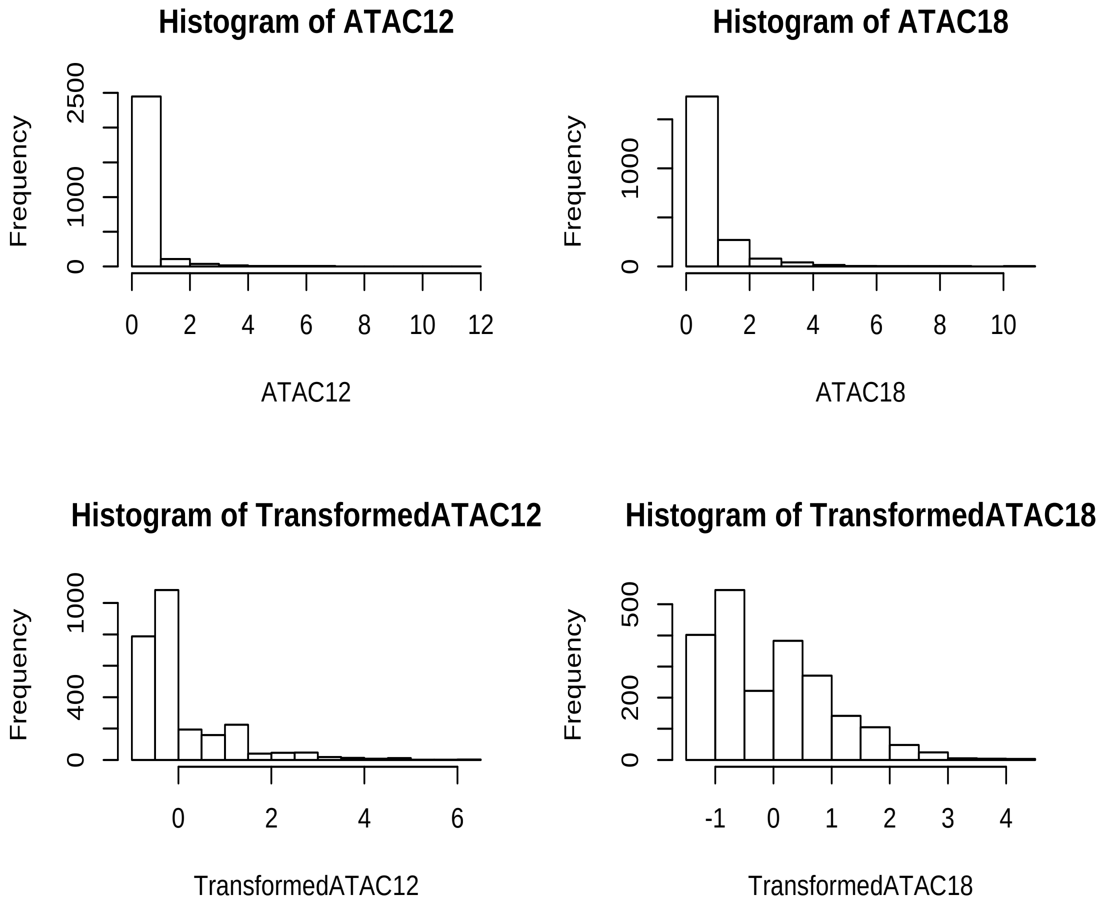

ATAC12: Untransformed Autism-Tics, AD/HD and other Comorbidities inventory, age 9/12
ATAC18: Untransformed Autism-Tics, AD/HD and other Comorbidities inventory, age 18
TransformedATAC12: Log transformed Autism-Tics, AD/HD and other Comorbidities inventory, age 9/12 (scale used in analyses)
TransformedATAC18: Log transformed Autism-Tics, AD/HD and other Comorbidities inventory, age 18 (scale used in analyses)
