# Supplementary material for: Etiological influences on the stability of autistic traits from childhood to early adulthood: evidence from a twin study
Source: Mol Autism. 2017 Feb 17;8:5. doi: 10.1186/s13229-017-0120-5 (PMC5351180; doi:10.1186/s13229-017-0120-5)
Supplement: Additional file 3: — Saturated model fitting. Table showing the results of saturated model fitting used to test assumptions and sex differences. (PDF 117 kb) [file 13229_2017_120_MOESM3_ESM.pdf]

Tests of saturated models

Model	-2LL	df	Parameters	AIC	BIC	$\Delta\chi^2$	Δdf	p
1	25044.31	9494	70	6056.31	-49743.70	-----	-----	-----
2	25265.38	9502	62	6261.38	-49585.65	221.207	8	<.001
3	25072.52	9502	62	6068.52	-49778.51	28.22	8	<.001
4	25067.06	9498	66	6071.06	-49752.46	22.75	4	<.001
5	25047.08	9498	66	6051.08	-49772.44	2.77	4	.60

Model descriptions:
1. Unconstrained saturated model
2. Equal variances across zygosity for autistic traits at age 9
3: Equal variances across zygosity for autistic traits at age 18
4: Equal cross-twin covariances across sex (allowing opposite-sex twin correlations to differ from same-sex twin correlations)
5: Equal cross-trait cross-twin covariances across sex (allowing opposite-sex twin correlations to differ from same-sex twin correlations)
The fit of the four constrained models is compared with the unconstrained model (model 1) in all cases
*-2LL: fit statistic, which is -2*log-likelihood of data; df: degrees of freedom; AIC: Akaike's Information Criteria; $\Delta\chi^2$: difference in -2LL between the unconstrained saturated model and each constrained model, distributed χ^2; Δdf: change in degrees of freedom between two models, equal to the difference in number of parameters; p: p-value from the likelihood-ratio test. Significant values (defined as p<.05) reflect that a given model fits significantly worse than the unconstrained model.*
