# Supplementary material for: Etiological influences on the stability of autistic traits from childhood to early adulthood: evidence from a twin study
Source: Mol Autism. 2017 Feb 17;8:5. doi: 10.1186/s13229-017-0120-5 (PMC5351180; doi:10.1186/s13229-017-0120-5)
Supplement: Additional file 4: — Description of DeFries-Fulker analytic method. Detailed outline of the DeFries-Fulker analytic procedure used to analyze extreme scoring groups. (PDF 266 kb) [file 13229_2017_120_MOESM4_ESM.pdf]

Description of DeFries-Fulker analytic method

DeFries-Fulker analysis is a regression-based analysis of means. In the univariate case, the procedure is concerned with estimating group heritability, the extent to which genetic influences on extreme scores in a trait also influence continuous variation in the same trait [1]. Bivariate analysis estimates bivariate heritability, which indexes the degree to which genetic influences on extreme scores on one trait influence continuous variation in scores in another trait [2]. Performing univariate and bivariate DeFries-Fulker analysis on two measures enables estimation of the genetic correlation between extreme scores on two traits [3]. The univariate and bivariate procedures are described separately below. Opposite-sex twins were not included in DeFries-Fulker analyses, since we had insufficient statistical power to examine sex differences in these analyses.

Univariate DeFries-Fulker Analysis

Extreme-scoring individuals, termed 'probands', were selected on the basis of scoring within the highest 5% of the A-TAC z-score distribution at age 9/12 or 18 (age 9/12 z-score = 2.46; age 18 z-score = 2.05). Initially, scores must be transformed so that the proband mean on the measure of interest is 1, while the control mean is 0. The mean score of co-twins of probands should then lie between 0 and 1. An initial indication of group heritability can be obtained by examining the co-twin means by zygosity; due to the genetic similarity of monozygotic (MZ) twins compared with dizygotic (DZ) twins, one would expect the DZ co-twin mean to regress towards zero to a greater degree than that of MZ twins if there is group heritability in a trait.

Group heritability was then formally estimated from the regression equation $C = B_1P+B_2R+K$. C is the co-twin score on the trait under consideration, B_1P represents the regression coefficient for proband scores, B_2R is the regression coefficient for zygosity, and K is the regression constant. B_2R is twice the difference between MZ and DZ transformed co-twin means, and thus is an estimate of group heritability, although this estimate was capped at the transformed co-twin mean for MZ twins.

Bivariate DeFries-Fulker Analysis

Bivariate DeFries-Fulker analysis is directional, in that one measure (the selection measure) is used to select probands while the other measure is the outcome measure. Thus, bivariate analysis was first performed using the A-TAC at age 9/12 as the selection measure and the A-TAC at age 18 as the outcome measure, and vice-versa.

Bivariate analysis started with the calculation of phenotypic group correlations, which highlight the degree of phenotypic association between two traits within an extreme scoring group. They were calculated by dividing the proband mean z-scores on the outcome measure by proband mean z-scores on the selection measure.

Bivariate DeFries-Fulker analyses were performed in scores that were transformed such that the mean proband score on the selection measure was 1, while the control mean for the outcome measure was 0. Co-twin means on the outcome measure were between 0 and 1 [4]. Examining these co-twin means by zygosity gives an initial indication of bivariate heritability, with bivariate heritability indicated when the DZ co-twin mean regresses towards 0 to a greater degree than the MZ co-twin mean. Bivariate heritability was then estimated from the

regression equation $C_y = B_1P_X+B_2R+K$. C_y represents co-twin scores on the outcome measure, B_1P_X is the regression coefficient for proband scores on the selection variable, B_2R is the regression coefficient for zygosity, and K is the regression constant. The regression of outcome measure co-twin scores on zygosity directly estimates bivariate heritability, which was again capped to at the transformed co-twin mean for MZ twins.

After performing univariate analysis for both measures and bivariate analyses in both directions, the genetic correlation between extreme A-TAC scores at each age was calculated from the equation $r_G = \sqrt{\frac{(B_2(xy))(B_2(yx))}{(B_2(x))(B_2(y))}}$, where B_2(xy) and B_2(yx) are the bivariate heritabilities and B_2(x) and B_2(y) are the group heritabilities.

Additional Considerations

Sex was included in all regression equations to control for the effect of sex on mean scores. Standard errors were adjusted following the analyses to account for the use of related individuals in regression analyses.

References

[1] DeFries JC, Fulker DW. Multiple regression analysis of twin data. Behav Genet. 1985;15:467-73.

[2] Light JG, DeFries JC. Comorbidity of reading and mathematics disabilities: genetic and environmental etiologies. J Learn Disabil. 1995;28:96-107.

[3] Knopik VS, Alarcon M, DeFries JC. Comorbidity of mathematics and reading deficits: evidence for a genetic etiology. Behav Genet. 1997;27:447-53.

[4] Purcell S, Eley TC, Dale PS, Oliver B, Petrill SA, Price TS, et al. Comorbidity between verbal and non-verbal cognitive delay in 2-year-olds: a bivariate twin analysis. Developmental Science. 2001;4:195-208.
