# Supplementary material for: Etiological influences on the stability of autistic traits from childhood to early adulthood: evidence from a twin study
Source: Mol Autism. 2017 Feb 17;8:5. doi: 10.1186/s13229-017-0120-5 (PMC5351180; doi:10.1186/s13229-017-0120-5)
Supplement: Additional file 5: — DeFries-Fulker analysis results. Three tables showing the full results of DeFries-Fulker analysis of the extremes, including descriptive statistics, univariate results, and bivariate results. (PDF 88 kb) [file 13229_2017_120_MOESM5_ESM.pdf]

**DeFries-Fulker Analysis Results**

*Table 1 Number of probands and descriptive statistics*

| Measure | N MZ Pro. | N DZ Pro. | Age 12 Mean | Age 18 Mean |
|---|---|---|---|---|
| ATAC 9/12 | 58 | 91 | 3.37 (1.90) | 2.59 (2.27) |
| ATAC 18 | 75 | 73 | 1.60 (2.22) | 4.08 (1.55) |

*A-TAC: Autism, tics, and other comorbidities inventory*
*N MZ Pro: number of MZ probands; N DZ Pro: number of DZ probands*
*Age 12 Mean: mean A-TAC score at age 12; Age 18 Mean; mean A-TAC score at age 18*

*Table 2 Univariate DeFries-Fulker analysis*

| | MZ Transformed Co-Twin Mean | DZ Transformed Co-Twin Mean | Group Heritability |
|---|---|---|---|
| A-TAC Age 9/12 | .59 | .16 | .59 (.28-.59) |
| A-TAC Age 18 | .69 | .11 | .69 (.36-.69) |

*Table 3 Bivariate DeFries-Fulker analysis*

| Analysis | PGC | Transformed Co-Twin Means | | Bivariate Heritability |
|---|---|---|---|---|
| | | MZ | DZ | |
| A-TAC 9/12 -> A-TAC 18 | .48 | .46 | .05 | .46 (.13-.46) |
| A-TAC 18 -> A-TAC 9/12 | .48 | .36 | .10 | .36 (-.01-.36) |

*A-TAC 9/12: Autism, tics, and other comorbidities inventory at age 9/12; A-TAC 18: Autism, tics and other comorbidities inventory at age 18*
*PGC: phenotypic group correlation; MZ: monozygotic twins; DZ: dizygotic twins*
